# Hippocampal CA1 Neurons Represent Positive Feedback During the Learning Process of an Associative Memory Task

**DOI:** 10.3389/fnsys.2021.718619

**Published:** 2021-09-06

**Authors:** Shogo Takamiya, Kazuki Shiotani, Tomoya Ohnuki, Yuma Osako, Yuta Tanisumi, Shoko Yuki, Hiroyuki Manabe, Junya Hirokawa, Yoshio Sakurai

**Affiliations:** ^1^Laboratory of Neural Information, Graduate School of Brain Science, Doshisha University, Kyoto, Japan; ^2^Research Fellow of the Japan Society for the Promotion of Science, Tokyo, Japan; ^3^Laboratory of Brain Network Information, College of Life Sciences, Ritsumeikan University, Shiga, Japan; ^4^Department of Life Sciences, Graduate School of Arts and Sciences, The University of Tokyo, Tokyo, Japan

**Keywords:** learning and memory, associative memory, recordings, reinforcement learning, hippocampus, rats

## Abstract

The hippocampus is crucial for forming associations between environmental stimuli. However, it is unclear how neural activities of hippocampal neurons dynamically change during the learning process. To address this question, we developed an associative memory task for rats with auditory stimuli. In this task, the rats were required to associate tone pitches (high and low) and ports (right and left) to obtain a reward. We recorded the firing activity of neurons in rats hippocampal CA1 during the learning process of the task. As a result, many hippocampal CA1 neurons increased their firing rates when the rats received a reward after choosing either the left or right port. We referred to these cells as “reward-direction cells.” Furthermore, the proportion of the reward-direction cells increased in the middle-stage of learning but decreased after the completion of learning. This result suggests that the activity of reward-direction cells might serve as “positive feedback” signal that facilitates the formation of associations between tone pitches and port choice.

## Introduction

The hippocampus plays a critical role in encoding spatial memory (O’Keefe and Nadel, 1978; McNaughton et al., 2006) and associative memory that associates olfactory (Eichenbaum et al., 1987), visual (Sakurai, 1996), and/or auditory (Sakurai, 1990, 1996) information in addition to spatial information. Associative memory becomes independent of hippocampal function when consolidated (Eichenbaum, 2000; Frankland and Bontempi, 2005). According to the two-stage model (Buzsáki, 1996, 2015), the hippocampus rapidly encodes information via changes in the synaptic strength during behavioral acquisition, and then the information is repeatedly replayed during slow-wave sleep and transferred to the neocortex. Recent studies utilizing optogenetics have revealed that reactivation of neurons in the hippocampus is necessary for retrieval of “recent” memory, while reactivation of neurons in the neocortex is necessary for retrieval of “remote” memory (Kitamura et al., 2017). However, optogenetic experiments used simple behavioral tasks using reflex responses that can be learned in a single experience (trial), such as contextual fear conditioning. Therefore, it is unclear how the neural activities of hippocampal neurons dynamically change during the learning process, in which associative memory is gradually modified from recent and unstable memory to stable one over a longer time span.

Hattori et al. (2015) addressed this exact question using chronic electrophysiology and trace conditioning of eyeblink reflex in rabbits. They revealed the learning-specific activity of hippocampal neurons in both the acquisition and retrieval of associative memories. The present study aimed to address the same question as that addressed by Hattori et al. (2015) but using operant behavioral conditioning with rewards. We developed an associative memory task for rats with auditory stimuli, as rodents have a high auditory acuity, and it is easier to regulate the difficulty of the task by modulating tone pitches. In this task, the rats were required to associate tone pitches (high and low) and ports (right and left) to obtain rewards. We recorded the firing activity of neurons in rat hippocampal CA1 during some days of the learning process of the task. We hypothesized that the number of task-related neurons in the hippocampal CA1 might increase when the rats were acquiring the task and decrease when they had learned it by memory consolidation.

## Materials and Methods

### Animals

Seven male Wister albino rats (Shimizu Laboratory Supplies, Kyoto, Japan) were individually housed and maintained on a laboratory light/dark cycle (lights on at 8:00 and off at 21:00). The rats were placed on food restriction with *ad libitum* access to water. The animals were maintained at approximately 80% of their baseline weight throughout the experiments. All experiments were conducted following the guidelines for the care and use of laboratory animals provided by the Animal Research Committee of Doshisha University.

### Apparatus

**FIGURE 1:**
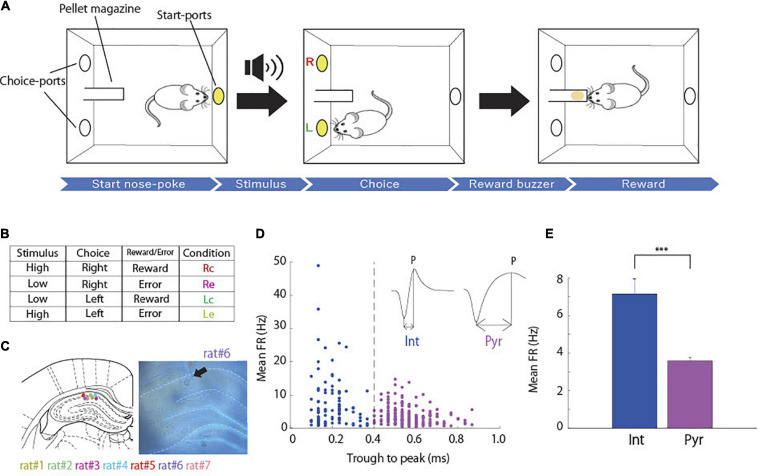
Associative memory task and cell classification. **(A)** Schematic representation of the task. **(B)** Table of task conditions, combinations of stimuli, choices and outcomes, and their abbreviations. **(C)** The coronal section indicating recording sites in hippocampal CA1 (arrow). Each color dots corresponds to the animal identification number. Modified from Paxinos and Watson (2007). **(D)** Classification of putative interneurons (Int, blue) and putative pyramidal neurons (Pyr, magenta) according to spike width (dashed lines). Spike width was measured by subtracting the time at the troughs from that at the peak of each spike waveform (arrow lines). **(E)** Comparison of mean firing rates between putative pyramidal neurons (*n* = 207) and interneurons (*n* = 99; two-sample *t*-test; *p* = 1.51 × 10^–8^) ***p < 0.001.

Behavioral training was performed in an operant chamber, 23 × 11 × 35 cm (Ohara-Ika, Tokyo, Japan), with two ports in the front wall and a port in the back wall for snout-poke response (Figure 1A). Each port was equipped with an LED light, which is an infrared sensor to detect nose-poke responses in the animal. A loudspeaker (15 cm in diameter) was placed 15 cm above the top of the chamber for sound stimuli. A food dispenser delivered a 45 mg food pellet to a magazine located 1.5 cm above the floor and on the middle of the front wall. The chamber was enclosed in a soundproof box (Brain Science Idea, Osaka, Japan). All events were controlled using a personal computer (NEC, Tokyo, Japan).

### Behavioral Task

Rats were trained in an associative memory task with tones, where they were required to associate tone pitches (high and low) and port locations (right and left). At the start of each trial, the port in the back wall was lighted on, and a high or low tone was randomly presented when the rat poked its snout into the port. Subsequently, the light of the port in the back wall turned off, and the right and left ports in the front wall were lighted on. When the tone was high, it was correct for the rat to poke its snout into the right port. When the tone was low, the correct response was to choose the left port. Immediately after the choice response, the light of the ports in the front wall turned off. A food pellet was delivered into the pellet magazine along with a buzzer noise when the rat made the correct choice (Figure 1A), and then the port in the back wall was lit again to start the next trial. When the rat chose incorrect ports, a time out was imposed, and the lighting on the back wall port to start the next trial was delayed for 5 s. When the rat did not choose either the right or left port for 10 s, the current trial was canceled.

Rats were trained with 1 and 3 kHz tone stimuli until the accuracy reached over 80%. After completion of the training, the rats underwent surgery for electrodes implantation. A week after the surgery, the rats were trained in the same task, but the tone stimuli were 6 and 10 kHz. They were trained until the choice accuracy was over 80%, and we recorded neural activity during the training process. Each training session consisted of 150–200 trials per day.

### Surgery

The surgical procedure was almost identical to that of previous studies (Ohnuki et al., 2020; Osako et al., 2021). Rats were anesthetized with 2.5% isoflurane before surgery and were maintained throughout the surgical procedure. We monitored the body temperature and depth of anesthesia as needed. An eye ointment was used to keep the eyes moistened throughout the surgery. A craniotomy was performed over the right hippocampal (AP, -3.2 to -3.0 mm, ML, 2.2 to 2.5 mm relative to the bregma, 1.5 mm below the brain surface), and custom-designed tetrodes attached to a microdrive were vertically implanted using a stereotactic manipulator. A stainless steel screw was placed over the cerebellum and served as the ground during recording.

### Recording

For each rat, eight tetrodes composed of four tungsten wires (12.5 μm, California Fine Wire, Grover Beach, CA, United States) were used for the extracellular recordings. Each tetrode was covered by a polyimide tube (A-M Systems, Sequim, WA, United States) and placed at a 100 μm separation. The tip impedance was 200–1,000 kΩ at 1 kHz. The signals were recorded using a head stage (Intan Technologies, United States) and a multichannel electrophysiology acquisition board (Open Ephys, Cambridge, MA, United States) at a sampling rate of 30 kHz and bandpass filtered between 0.3 and 6 kHz. The mean activity of all tetrodes was used as a reference. During a week of postsurgical recovery, the tetrodes were advanced by 20 μm per day until firing from some cells was observed. We did not move the tetrodes during the training of the rats to record the same cell population throughout the training process.

### Histology

After the experiment, each rat was anesthetized with sodium pentobarbital and perfused with phosphate-buffered saline and 4% paraformaldehyde. The brain was removed and post-fixed in 4% paraformaldehyde, and 50 μm coronal sections of the brain were prepared to confirm the recording sites.

### Data Analysis

Spike sorting analyses were performed using MATLAB (MathWorks, Natick, MA, United States). To detect single neuron activity, the spikes were manually clustered with MClust (A.D. Redish) in MATLAB. Only neurons that met the following criteria were included for further analyses: (1) spikes with sufficient isolation quality (isolation distance ≥ 15); (2) spikes with reliable refractory periods (violations were less than 1% of all spikes).

Detecting task-related neurons: To evaluate the task-related neurons, we computed peri-stimulus time histograms using a 20 ms bin width and smoothed by convolving spike trains with a 40-ms wide Gaussian filter in four trial outcome conditions (Rc, right-correct; Re, right-erroneous; Lc, left-correct; and Le, left-erroneous; Figure 1B). For each neuron, we performed a sliding ROC analysis (Shiotani et al., 2020; Tanisumi et al., 2021). Starting from the baseline period of each condition (4 s of fixation before trial start), an ROC value was calculated for a 100 ms bin. This bin was then stepped forward in 20 ms increments until the time after 4 s from the choice epoch. We also performed area under the receiver operating characteristic (auROC) analysis to evaluate the selectivity of each neuron by comparing the firing rate of each trial condition (Rc *vs.* Re, Rc *vs.* Le, Rc *vs.* Lc, Lc *vs.* Le, and Le *vs.* Re) in the same way as aforementioned. To determine the statistical significance (*p* < 0.05), we used permutation tests (1,000 iterations). We defined several task-related neurons under the following conditions: “Choice-direction cell:” (1) auROC values of the Rc or Lc that were calculated by comparing the firing rates of each period to the baseline of each condition were significant for five bins in a row in the period from the trial started to the points of choice response; (2) auROC values of Rc *vs.* Lc that were calculated by comparing the firing rates of the Rc trials to those of the Lc trials were significant for the same periods as (1). “Reward-direction cell:” (1) either the auROC values of the Rc trials or that of the Lc trials that were calculated by comparing the firing rates of each period to the baseline of each condition were significant for five bins in a row in the period from choice response to the time after 3 s from choice response; (2) auROC values of the Rc (Lc) trials that were calculated by comparing the firing rates of the Rc (Lc) trials to that of the other trials were significant for the same periods as (1). “Choice-reward-direction cells:” The cells that met both conditions of choice-direction cells and reward-direction cells.

Quantifying the degree of selectivity to directions: To evaluate the selectivity to different directions of choice in choice and reward epochs, we computed selectivity index using mean absolute auROC values of Rc *vs*. Lc in each epoch.

## Results

We recorded the spiking activity of 306 hippocampal CA1 cells from rats while they were learning the task (Figure 1C). Neurons were categorized as putative pyramidal cells and interneurons based on their spike width (Isomura et al., 2009; Figure 1D). We obtained 207 putative pyramidal neurons and 99 putative interneurons that showed significantly higher average firing rates than putative pyramidal neurons (Figure 1E; two-sample *t*-test, *p* = 1.51 × 10^–8^).

**FIGURE 2:**
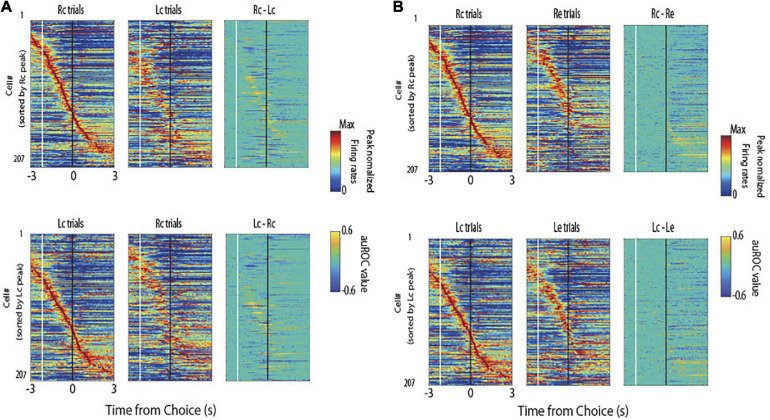
Normalized peak firing rates and area under the receiver operating characteristic (auROC) values of all pyramidal neurons. **(A)** Firing patterns across all pyramidal neurons (*n* = 207) for the right correct (Rc) and left correct (Lc) trials. In each trial type, the mean firing rate of each neuron was normalized to its peak (left and middle panels), and auROC values were calculated by comparing the firing rates of Rc trials to those of Lc trials (upper) and Lc trials to those of Rc trials (lower). These neurons were sorted by their peak firing time in Rc trials (upper) and Lc trials (lower). White and black lines indicate the times of tone stimulus on and choice response, respectively. The right panels represent the firing rates subtracting Lc trials from Rc trials (upper) and Rc trials from Lc trials (lower). **(B)** Firing patterns across the same neurons for the Rc and right erroneous (Re) trials (upper) and Lc and Le trials (lower) are shown as in panel **(A)**.

Figure 2 shows the firing patterns across all pyramidal neurons (*n* = 207) for the four task conditions (Figure 1B). Many choice-selective cells showed activity with significant differences in the auROC curve values between the Rc and Lc trials during the stimulus epoch. The upper portion of Figure 2A shows the cells sorted by their peak firing time in the Rc trials and compared the Rc *vs.* Lc trials during the stimulus and reward epochs. The lower portion sorts the cells by their peak firing time in the Lc trials and compared the Lc *vs.* Rc trials. Many reward-selective cells showed activity with significant differences between c (correct) and e (erroneous) trials during the reward epoch (Figure 2B). The upper portion of Figure 2B shows the cells sorted by their peak firing time in Rc trials and compared Rc *vs*. Re trials. The lower portion sorted the cells by their peak firing time in the Lc trials and compared the Lc *vs.* Le trials.

**FIGURE 3:**
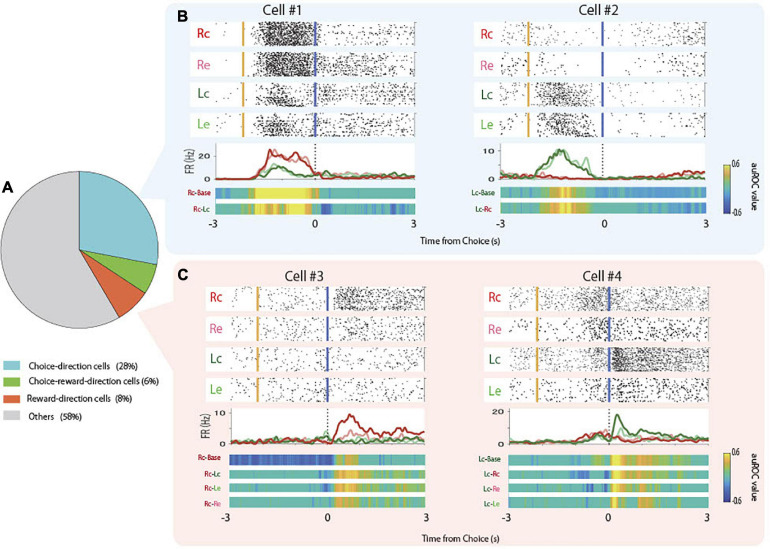
Proportions and examples of choice-direction cells and reward-direction cells. **(A)** Proportion of choice-direction cells, reward-direction cells, and choice-reward-direction cells in the total pyramidal neurons. **(B)** Examples of firing patterns of choice-direction cells showed firing increments in Rc trials (Cell #1) and Lc trials (Cell #2). Top panels: raster plots for each task condition, yellow and blue lines indicate the times of tone stimulus on and choice response, respectively. Middle panels: PSTHs of Rc trials (red), Re trials (pink), Lc trials (green), and left erroneous (Le) trials (light green). Dashed lines indicate the times of the choice response. Bottom panels: auROC values of task conditions to be defined as choice-direction cells (permutation test, *p* < 0.05). **(C)** Examples of firing patterns of reward-direction cells that showed firing increments in Rc trials (Cell #3) and Lc trials (Cell #4) are presented as in panel **(B)**.

Some pyramidal neurons increased firing rate prior to the choice of specific direction regardless of whether the choice was correct or not (choice-direction cells, Figure 3B, Cell #1, #2). In parallel, other cells increased firing rate after the reward delivery following the choice of specific direction when the choice was correct (reward-direction cells, Figure 3C, Cell #3, #4). We observed that 34% of pyramidal neurons were choice-direction cells or choice-direction and reward-direction cells (“choice-reward-direction cells”; Figure 3A). We also observed that 14% of pyramidal neurons were reward-direction cells or choice-reward-direction cells (Figure 3A). Only 4% of pyramidal neurons responded to reward regardless of the choice direction and 8% of pyramidal neurons responded to reward omission (error). We observed that 9 and 14% of interneurons were choice-direction/choice-reward-direction cells and reward-direction/choice-reward-direction cells, respectively.

**FIGURE 4:**
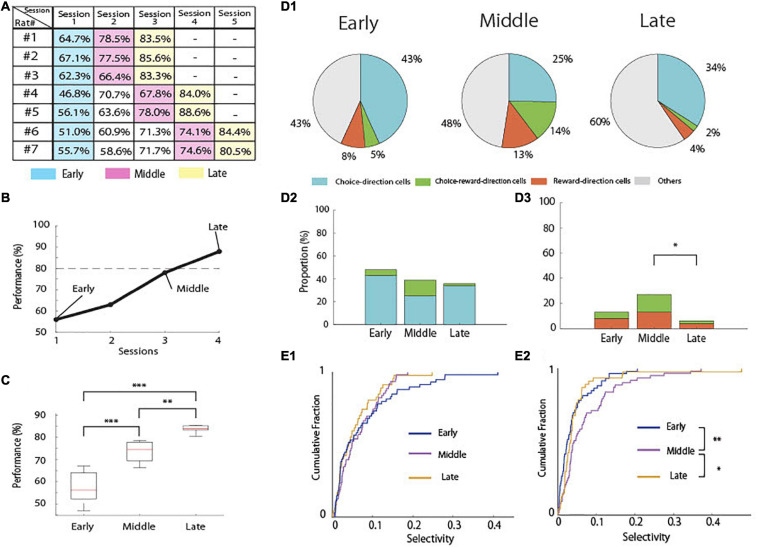
Behavioral learning stages and proportion of task-related cells. **(A)** An example of performance in the associative memory task (rat #1). The dashed line indicates 80% correct. **(B)** Performance accuracy of the task in each rat in each session. **(C)** Mean correct performance in the task in each learning stage in all rats (*n* = 7). **(D1)** Proportion of task-related cells in each learning stage. **(D2)** Comparison of the proportions of choice-direction cells including choice-reward-direction cells among the learning stages (Chi-square test and Fisher’s exact test with Holm correction). **(D3)** Comparison of the proportions of reward-direction cells including choice-reward-direction cells among the learning stages (Chi-square test and Fisher’s exact test with Holm correction). **(E1)** Cumulative density plots comparing the selectivity of choice-direction cells including choice-reward-direction cells among the learning stages in choice epoch. **(E2)** Cumulative density plots comparing the selectivity of reward-direction cells including choice-reward-direction cells among the learning stages in reward epoch (Kruskal–Wallis test and Multiple comparison with LSD test). ***p < 0.001, **p < 0.01, and *p < 0.05.

The rats used in the present study acquired the task in three to five sessions (days; Figure 4A). To assess the changes in neural activity by learning, we separated the total learning sessions into three stages (Figure 4B). We defined the first session as the “early-stage” (mean behavioral accuracy = 57.7%, *n* = 7) and the last session as the “late-stage” (mean behavioral accuracy = 84.29%, *n* = 7). We also defined the sessions before the last session as the “middle-stage” (mean behavioral accuracy = 73.85%, *n* = 7). The behavioral accuracies of these three groups were significantly different (Figure 4C; one-way ANOVA: *F*(2,18) = 44.39, *p* = 1.09 × 10^–7^; Tukey’s HSD: Early < Middle, *p* = 6.2 × 10^–5^, Middle < Late, *p* = 0.0047, Early < Late, *p* = 7.31 × 10^–8^).

We then compared the proportions of task-related pyramidal neurons in the early (*n* = 60), middle (*n* = 63), and late stages (*n* = 47; Figure 4D1). The tetrodes were not moved, and constant spike waveforms of individual neurons were confirmed during recording, suggesting that the recordings were very likely from the same cell population. However, occasionally some neurons disappeared or new neurons appeared during the recording (Li et al., 2017), resulting in different numbers of recorded neurons among the three stages of learning.

For the choice-direction-cells, we observed no significant differences among the learning stages (Figure 4D2). However, the proportion of the reward-direction cells including choice-reward-direction cells was significantly different among the learning stages (Figure 4D3; chi-square test, χ^2^(2) = 8.9672, *p* < 0.025). The proportion was 27% in the middle-stage, but it significantly decreased to 6% in the late-stage (Fisher’s exact test with Holm correction; *p* = 0.0176).

We also analyzed all data to quantify the degree of selectivity in each neuron using auROC and compared the distribution of these measures between the three learning stages (Figures 4E1,E2). The results of the proportions of task-related neurons (Figures 4D1–D3) were confirmed by the results of the degrees of selectivity in the neurons (Figures 4E1,E2). Although no significant difference was found among the learning stages in choice epoch (Figure 4E1), the degrees of selectivity differed significantly among the learning stages in reward epoch (Figure 4E2, Kruskal–Wallis test, *p* = 0.004). The selectivity in the middle stage significantly higher than those in the early and late stages (Multiple comparison with LSD test; Early < Middle, *p* = 0.0013, Late < Middle, *p* = 0.0482).

## Discussion

In this study, we report the neuronal activity in the hippocampal CA1 during the entire process of learning an auditory associative memory task. We found that several pyramidal neurons showed choice-direction selective (Figures 3A,B) or reward-direction selective (Figures 3A,C) activity. The property of choice-direction cells might be the association between cue tone and choice (Terada et al., 2017) or goal-directed encoding (Aoki et al., 2019; Igata et al., 2021). However, the proportion of the choice-direction cells was not learning-dependent and did not significantly differ among the learning stages (Figure 4D2), suggesting that their firing might reflect a stable function in hippocampal CA1 throughout the learning process, such as spatial coding of choice and/or ports.

For the reward-direction cells, we observed that their proportion was learning-dependent (Figures 4D3,E2) and significantly decreased in the late stage of the learning process, although the rats received the highest amount of reward at the last stage. It is obvious that the reward-direction cells do not represent reward delivery itself because of their learning-dependent property. Previous studies have revealed that some hippocampal neurons represent reward-predicted encoding and called such neurons “reward cells” (Gauthier and Tank, 2018). However, in our study, reward-direction cells did not represent reward-predicted encoding or reward locations because these cells were activated after the rats chose the correct port and reward buzzer was presented, and reward pellets were delivered into the same location (pellet magazine in Figure 1A) irrespective of the location of the correct port. Therefore, the firing of reward-direction cells might reflect “positive feedback” of the correct port choice to form the association of auditory stimuli and port directions. They play a role in reinforcement learning only when the learning is not completely acquired as their selectivity significantly increased in the middle state (Figure 4E2) and their selectivity and proportion significantly decreased in the late stages of learning (Figures 4D3,E2). In reinforcement learning, positive feedback is crucial to acquire learning tasks (Maia, 2009; Littman, 2015), and the reward-direction cells in CA1 might underlie the positive feedback to make the progress of learning. However, after completion of learning, the activation of these CA1 cells might become unnecessary as learned information is transferred to the neocortex for memory consolidation.

Although it is unclear whether associative memory and reinforcement learning rely on a common neural substrate in the hippocampus, it may be useful to discuss the present data of reward-direction cells from a reinforcement learning perspective, e.g., the idea of the successor representation (SR; Stachenfeld et al., 2017; Gershman, 2018). Besides that the SR is closely related to place fields observed in the hippocampal CA1 (Stachenfeld et al., 2017), it takes on richer characteristics in other complex environments. If the hippocampus encodes the SR, then we can predict how it will respond to transition and reward manipulations in revaluation experiments (Momennejad et al., 2017). What seems to be examined, particularly in relation to the positive-feedback signals of the reward-direction cells, is whether the cells convey vector-valued signals to update the SR (Gershman, 2018). Ensemble recordings of reward-related cells will be useful in answering this question.

The reward-direction cells that have a joint feature of reward state and direction seem to be relevant to the multidimensional features of hippocampal neurons (Nieh et al., 2021). Nieh et al. (2021) examined how neurons in the CA1 integrated neural representations of cognitive and physical variables and whether low-dimensional manifolds underlie these representations. They found that the majority of task-related neurons encoded position and evidence jointly in the multidimensional spaces and suggest that the neural encoding of the task variables at the cellular level may have a geometric structure. In a future experiment following the present study, it is necessary to examine how the multidimensional activities of reward-direction and choice-direction cells in the CA1 are integrated into neural representations of the variables of the present associative memory task and whether such an integration creates a task-specific structure.

Since the pioneering study by Olds et al. (1972), many studies have investigated neural activity changes during learning processes (e.g., McEchron and Disterhoft, 1997; Wirth et al., 2003; Igarashi et al., 2014; Modi et al., 2014). McEchron and Disterhoft (1997) reported that CA1 pyramidal neurons demonstrated changes in activity after the CS and/or US in different learning stages of trace eyeblink conditioning. Modi et al. (2014) found that CA1 neurons transiently increased their spontaneous activity correlations during trace eyeblink conditioning, and the correlated neurons fell into distinct spatial clusters that changed as a result of learning. Wirth et al. (2003) reported that hippocampal neurons signaled the acquisition of new associations by changing their stimulus-selective response properties. Igarashi et al. (2014) identified the entorhinal–hippocampal coupling by 20–40-Hz oscillations as a key mechanism for the formation and retrieval of associative memory.

These previous studies reported cue-evoked firing patterns and their changes of hippocampal CA1 neurons with learning, and have yielded extensive information about their role in the learning. In contrast, the present study shows learning-related changes in reward-evoked activity. We demonstrated an increment in the activity of reward-direction cells in CA1 at the middle stage of learning. Therefore, the present study might further reveal the role of hippocampal CA1 neurons in the learning of associative memory by suggesting that CA1 pyramidal neurons are working to provide the animal with positive feedback regarding the correct association to acquire the associative memory.

The results of our study indicate that hippocampal CA1 neurons play a role in acquisition of the association between auditory cue and direction choice. However, there is no evidence yet that the present memory task depends on the hippocampus and that memory retrieval becomes independent from the hippocampus. Further studies are required to reveal the hippocampal dependency of the present task in ablation or inactivation studies and, in particular, to reveal causality between the activation of reward-direction cells and learning progress in an optogenetic study.

## Data Availability Statement

The raw data supporting the conclusion of this article will be made available by the authors, without undue reservation.

## Ethics Statement

The animal study was reviewed and approved by the Animal Research Committee of Doshisha University.

## Author Contributions

ST and YS designed the experiments. ST performed the experiments and analyzed the data. KS, TO, YO, YT, SY, HM, JH, and YS supervised the project. All authors contributed to writing the manuscript and approved the submitted version.

## Conflict of Interest

The authors declare that the research was conducted in the absence of any commercial or financial relationships that could be construed as a potential conflict of interest.

## Publisher’s Note

All claims expressed in this article are solely those of the authors and do not necessarily represent those of their affiliated organizations, or those of the publisher, the editors and the reviewers. Any product that may be evaluated in this article, or claim that may be made by its manufacturer, is not guaranteed or endorsed by the publisher.
